# First detection of tick‐borne encephalitis virus (TBEV) in raw milk samples in North‐Western Iran

**DOI:** 10.1002/vms3.1477

**Published:** 2024-06-19

**Authors:** Angineh Parsadanians, Hessam Mirshahabi, Masoud Yavarmanesh

**Affiliations:** ^1^ Department of Microbiology and Virology Faculty of Medicine Zanjan University of Medical Sciences Zanjan Iran; ^2^ Department of Food Science and Technology Faculty of Agriculture Ferdowsi University of Mashhad Mashhad Iran

**Keywords:** raw milk, subtyping, Iran, nested‐reverse transcription‐polymerase chain reaction, tick‐borne encephalitis, zoonotic infection

## Abstract

Tick‐borne encephalitis virus (TBEV) is a significant cause of flaviviral infections affecting the human central nervous system, primarily transmitted through tick bites and the consumption of unpasteurized milk. This study aimed to assess the prevalence of TBEV and identify new natural foci of TBEV in livestock milk. In this cross‐sectional study, unpasteurized milk samples were collected from livestock reared on farms and analysed for the presence and subtyping of TBEV using nested reverse transcription‐polymerase chain reaction , alongside the detection of anti‐TBEV total IgG antibodies using ELISA. The findings revealed that the highest prevalence of TBEV was observed in goat and sheep milk combined, whereas no TBEV was detected in cow milk samples. All identified strains were of the Siberian subtype. Moreover, the highest prevalence of anti‐TBEV antibodies was detected in sheep milk. These results uncover new foci of TBEV in Iran, underscoring the importance of thermal processing (pasteurization) of milk prior to consumption to mitigate the risk of TBEV infection.

## Graphical abstract

New natural foci of TBEV were identified for the first time in Iran. The highest TBEV prevalence was observed equally in goat and sheep milk, with no detection in cow milk, whereas all identified strains belonged to the Siberian subtype. The highest prevalence of anti‐TBEV antibodies was found in the milk of sheep.

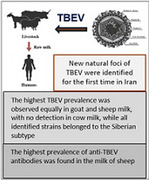

## 1 INTRODUCTION

Tick‐borne encephalitis (TBE) is a zoonotic viral disease caused by TBE *Flavivirus* (TBEV). The initial comprehensive depiction of the disease entity emerged in 1931 from Austria described by Schneider (1931). The virus, on the other hand, was initially isolated from human blood and the *Ixodes* ticks as vector in 1937 in Russia (Neitz & Du Toit, 1938; Shin, 2023). In nature, TBEV is transmitted between ticks and small rodents, serving as the primary reservoirs (Jääskeläinen et al., 2016). Despite Iran's notable diversity of rodent species and hard ticks, the prevalence of the virus and its vectors remains poorly documented in the country (Khoobdel et al., 2021; Paquette et al., 2023). When a tick is infected, it will remain infected and can transmit the virus and cause TBE infection until the end of its life (Ijaz et al., 2023). TBE cases exhibit various clinical symptoms in humans, such as headache, high fever, vomiting and chronic or acute progressive encephalitis, with or without fatal consequences (Shin, 2023). Three main subtypes of TBEV are recognized: the European subtype, spread by *Ixodes ricinus* ticks across Central Europe; and the Siberian (TBEV‐Sib) and Far‐Eastern (TBEV‐FE) subtypes, carried by *Ixodes persulcatus* ticks, extending from the far east to the Baltic countries (Pustijanac et al., 2023; Ruzek et al., 2019). Although two new subtypes, namely the Baikalian (TBEV) and Himalayan (TBEV) subtypes, have been recently identified, they were previously regarded as divergent TBEV strains, divided from Sib‐TBEV (Dai et al., 2018; Phipps & Johnson, 2022). The strains of the Sib subtype pertained more closely to those of the FE subtype than the European one. Consequently, it was supposed that the viruses of the Sib and FE subtypes had diverged later than the European strains (Kovalev & Mukhacheva, 2017). The severity of the disease resulting from these subtypes varies between Asia and Europe, as the clinical course and outcomes differ for each TBEV subtype. Specifically, the illness associated with the European subtype exhibits milder symptoms and more favourable clinical outcomes compared to infections caused by the FE and Sib subtypes (Baasandavga et al., 2019; Fortova et al., 2023). This may be happening because of virulence differences between these subtypes. Several studies show that the FE and Sib subtypes are more rigorous than the European one. Still, other factors, such as differences in hospitalization rates and the quantities of mild cases, might play a role (Ecker et al., 1999; Mazanik, 2023; Tykalová, 2022).

Transmission of the disease through milk, differing from tick bites, leads to conditions known as monophasic or biphasic milk fever (Pustijanac et al., 2023). This condition typically begins after a shorter incubation period of 3–4 days, compared to the 7–14 days associated with tick bite (Bogovic & Strle, 2015; Slavka et al., 2023). Nearly half of the cases exhibited a monophasic form of the disease, while the remainder presented with a biphasic illness characterized by more severe symptoms. The first phase, lasting about 7 days, involved visual disturbances or blurred vision and higher fever, followed by signs of meningitis and encephalitis in the second phase (Paraličová et al., 2022).

As the demand for raw milk grows, so does the potential for alimentary transmission of TBEV (Friker et al., 2020; Ličková et al., 2022). Although infected animals can produce TBEV‐containing milk, there has been no investigation into the presence of TBEV in raw milk in Iran for the first time to the best of our knowledge. Notably, the first seroprevalence study in healthy humans was conducted near Mazandaran province, Iran, 2 years after our research was carried out (Salehi‐Vaziri et al., 2020). The objective of this research was to determine the prevalence of TBEV and identify new natural foci in raw milk collected from different livestock animals in seven northwest regions of Iran. Our study made use of serological and molecular methods to detect the presence of TBEV in raw milk, demonstrating the efficacy of both techniques in identifying the infection.

## 2 MATERIALS AND METHODS

### 2.1 Sample collection and processing of milk

**FIGURE 1:**
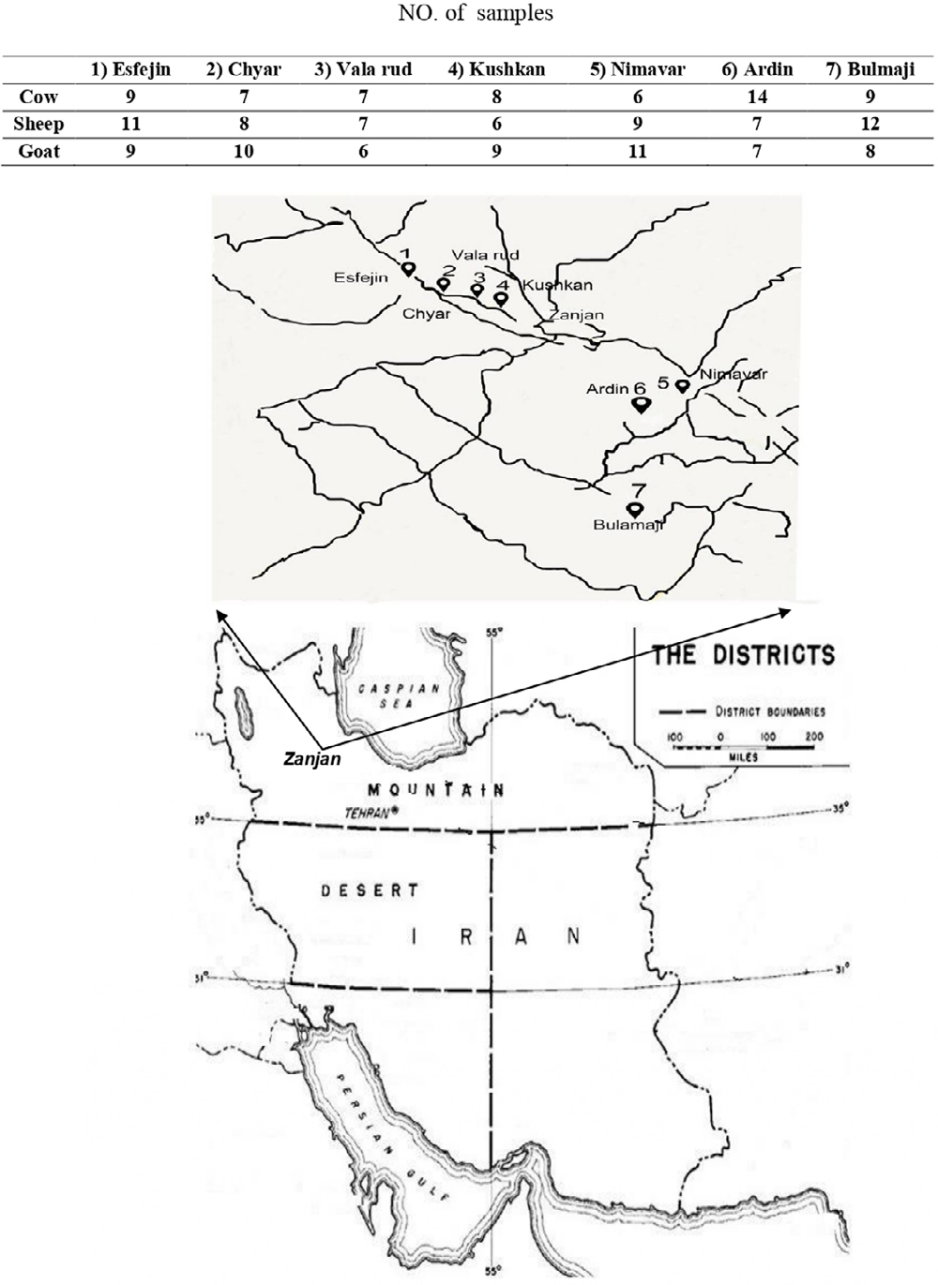
Milk collection sites distribution. Descriptions are summarized in Table 1.

One hundred eighty milk samples were collected from dairy goats, cows and sheep from various sampling sites positioned in two geographically distinct regions (Figure 1, Table 1) of the Zanjan province in 2016. The northern region (A) is arid and forested, and the western one (B) mostly spreads over forested and mountainous lands. For the purposes of this cross‐sectional study, milk samples were diligently collected from each of the seven sampling sites during the spring and summer. Each site received a single visit on a specific day for sample collection, with the entire sampling process taking place from May to August 2016. Sampling sites were selected from each region according to a stratified random sample collection strategy. Livestock owners agreed to participate in the study; voluntarily, milking was done traditionally in the shaded grazing field in front of the homestead or under trees.

**TABLE 1:** Total number of milk samples collected at each site and milking domesticated animals.

Sampling region	Sampling site name and number	Sampling season	Sampling actual date	GPS coordinates of the sampling sites	Total number of cow milk samples collected from each sampling site	Total number of sheep milk samples collected from each sampling site	Total number of goat milk samples collected from each sampling site
A. Northern part of the Zanjan province	Esfejin	August 2016	08.03.2016	36°45′46.5″N	9	11	9
			08.20.2016	48°14′48.8″E			
	Chiyar	July 2016	07.02.2016	36°44′59.0″N	7	8	10
			07.11.2016	48°18′16.5″E			
	Valaroud	May 2016	05.07.2016	36°43′31.8″N	7	7	6
			05.18.2016				
			05.28.2016	48°21′54.0″E			
	Koushkan	August 2016	10.11.2016	36°43′00.1″N	8	6	9
			10.29.2016	48°23′52.6″E			
B. Western part of the Zanjan province	Nimavar	June 2016	06.04.2016	36°34′41.8″N	6	9	11
			06.12.2016	48°40′34.3″E			
	Ardin	July 2016	07.21.2016	36°34′02.7″N	14	7	7
			07.30.2016	48°36′46.9″E			
	Bulamaji	June 2016	06.10.2016	36°23′18.8″N	9	12	8
			06.29.2016	48°34′58.2″E			
In total	7	Spring–summer			60	60	60

All milk samples were promptly collected into 50 mL Falcon test tubes right after milking. They were then transported in ice‐packed cooler boxes to the laboratory and examined within 24 h of collection. To break down the milk fat mechanically; the milk samples were centrifuged at 15,000 × *g* for 5 min at 4°C. A sampler tip was placed beneath the cream layer in the tube and the skim milk. Next, medial layer was transferred to a 2 mL microtube. Before polymerase chain reaction (PCR) and ELISA assays, skim milk samples were stored at −80°C.

### 2.2 Ethical considerations

This study received ethical approval from the Ethics Committee at XXX University of Medical Sciences, XXX, Iran, under the Approval Number IR.ZUMS.REC. A‐11‐864‐5.

### 2.3 RNA isolation

Using Qiamp Viral Mini Kit (Qiagen), total RNA was extracted from 140 μL of defatted milk according to the manufacturer's instruction between May 2016 and August 2016. The viral RNA was kept at −80°C for further analyses. The purity and concentration of the RNA extracts were determined using NanoDrop 1000 (Thermo Fisher Scientific Inc.). The RNA concentration was in the range of 46.7–134.2 ng/μL.

### 2.4 Quantitative reverse transcription‐polymerase chain reaction (RT‐qPCR)

The Quanti‐Tect reverse transcription (RT) Kit (Qiagen) was employed for RT‐PCR based on the manufacturer's instruction. The reaction volume was equal to 20 μL, which was comprised of the following mixture of reagents: RNase‐free water, gDNA Wipeout Buffer 7X, Quantiscript Reverse Transcriptase, Quantiscript RT Buffer 5X, RT primer mix from the 5′‐terminal noncoding region (Schrader & Süss, 1999) and template RNA at 2 μL. The reaction was conducted in Analytik Jena Thermal Cycler in the following conditions: gDNA elimination at 42°C for 2 min and RT at 42°C for 30 min and at 95°C for 3 min. The first PCR reaction was performed in a 25 μL volume composed of the following mixture of reagents: 1 × PCR buffer with 1.5 mM MgCl_2_, 0.2 U of Amplicon Taq DNA polymerase, 0.4 mM of each dNTP (all from Ampliqon), primers (Table 2) in a final concentration of 10 pmol of each (Metabion), 2 μL of cDNA and nuclease‐free water. The reaction was carried out under the following conditions: denaturation as the initial step at 94°C for 2 min, followed by 40 PCR cycles, each of 30 s at 94°C, 30 s at 55°C and 1 min at 72°C. The last extension was carried out at 72°C for 10 min.

**TABLE 2:** Primers used for the detection of tick‐borne encephalitis virus (TBEV) RNA and defining subtypes in raw milk samples.

Primer name		Sequence (5′ → 3′)	Genome position	Length	Reference
Pp1		GCG TTT GCT TCGGAC AGC ATT AGC	21–44	191	Hay et al. (2016)
Pm1		GCG TCT TCG TTGCGG TCT CTT TCG	188–211		
Pp2		TCG GAC AGCATT AGC AGC GGT TGG	30–53	178	
Pm2		TGC GGT CTCTTT CGA CAC TCG TCG	178–201		
European	E(F)	ACA CGG GAG ACT ATG TTG CCG CA	1409–1660	198	Henningsson et al. (2016)
	E(R)	CCG TTG GAA GGT GTT CCA CT			
Siberian	S(F)	GTG GAT GTG TCA CGA TCA CT	1057–1601	553	
	S(R)	GCC GTC GGA AGG TGT TCC AGA			
Far Eastern	FE(F)	TGG AGC TTG ACA AGA CCT CA	1578–2347	785	
	FE(R)	TCC CAC TAG GAT CTT GGG CAA			

### 2.5 Nested PCR

The second amplification was conducted with 2 μL of the first amplification product. The total reaction volume of 50 μL consisted of the following reagents: 1× PCR buffer containing 1.5 Mm MgCl_2_, 0.4 U of Amplicon Taq DNA polymerase, 0.4 mM of each dNTP (all from Ampliqon), primers (Table 2) concentration of 20 pmol each (Metabion) and nuclease‐free water. An Analytik Jena Thermal Cycler was utilized for the reaction under the same conditions as the first amplification, followed by 30 cycles to confirm positive sample identities. A QIAquick purification kit (QIAGEN) was employed to purify the PCR products, which were subsequently cloned into the pGEM‐T cloning system as control positive (Promega). Each plasmid clone was purified using a Purelink Quick Plasmid Miniprep Kit (Invitrogen). A water sample was employed as the negative control in every 0.2 microtube. The entire control samples were negative, which revealed there was no contamination during the PCR. PCR product of 6 μL was aded onto 2% agarose gel, run at 80 V for 35 min, and detected in UV light using a marker on 2% agarose gel.

### 2.6 Subtyping

PCR amplification was done based on the paper by Růžek et al. (2007) with some modifications. Using the 1× PCR buffer containing 1.5 Mm MgCl_2_, 0.2 U of Amplicon Taq DNA polymerase and 0.4 mM of each dNTP (all from Ampliqon), primers from the viral envelope (E) protein (Růžek et al., 2007) (Table 2) in a final concentration of 10 pmol of each (Metabion), nuclease‐free water and 2 μL of cDNA in the final volume of 25 μL. The cycling conditions included denaturation at 95°C for 5 min, followed by 35 cycles at 95°C for 30 s, 57°C for 40 s and 72°C for 90 s. The results were visualized by carrying out 2% agarose gel electrophoresis in Tris‐acetate–EDTA buffer.

### 2.7 DNA sequencing

All the positive products of RT‐PCR were purified using the QIAquick Gel extraction Kit (QIAGEN) based on the instruction presented by the manufacturer. After purification, the results were cloned into apCRTM4‐TOPO plasmid (Invitrogen) for confirmation. Sequencing was conducted by T7 promotor (5‐TAATACGACTCACTATAGGG‐3) and M13R‐pUC (5‐CAGGAAACAGCTATGAC‐3) primers using an ABI Prism BigDyeTMv3.1 Terminator Cycle Sequencing Kit and an ABI 3730xl Sequencer (Applied Biosystems) at Macrogen Inc. The sequencing results were aligned with the help of SeqMan PRO, Lasergene version 5.0.6 (DNASTAR Inc.). The sequences were compared to those published in the GenBank database using the BLAST server at the National Centre for Biotechnology Information (Bethesda).

### 2.8 ELISA

We used the commercial test Immunozym FSME IgG All Species (Progen Biotechnik GMBH) to detect the specific anti‐TBEV antibodies in the milk samples, which had been previously diluted with 0.1 M Tris/HCl buffer (pH = 7.4) at a ratio of 1:1 before the test, according to the study conducted by Cisak et al. (2010). The results were measured spectrophotometrically at an optical density of 450 nm. The manufacturer of the test kits provided the cut‐off values and borderline zones. Applying the procedure described in the kit manual, the experimental values of 63 and 126 VIEU/mL were confirmed as the lower and upper limits of a borderline zone, respectively. The sensitivity and specificity of the test outside the borderline zone were 97% and 99%, respectively. All positive samples showed results above 126 VIEU^i^/mL.

### 2.9 Statistical analysis

A descriptive statistic was used to summarize and organize characteristics of data set so that the number of TBEV‐positive raw milk samples and their frequency distribution were reported.

## 3 RESULTS

**FIGURE 2:**
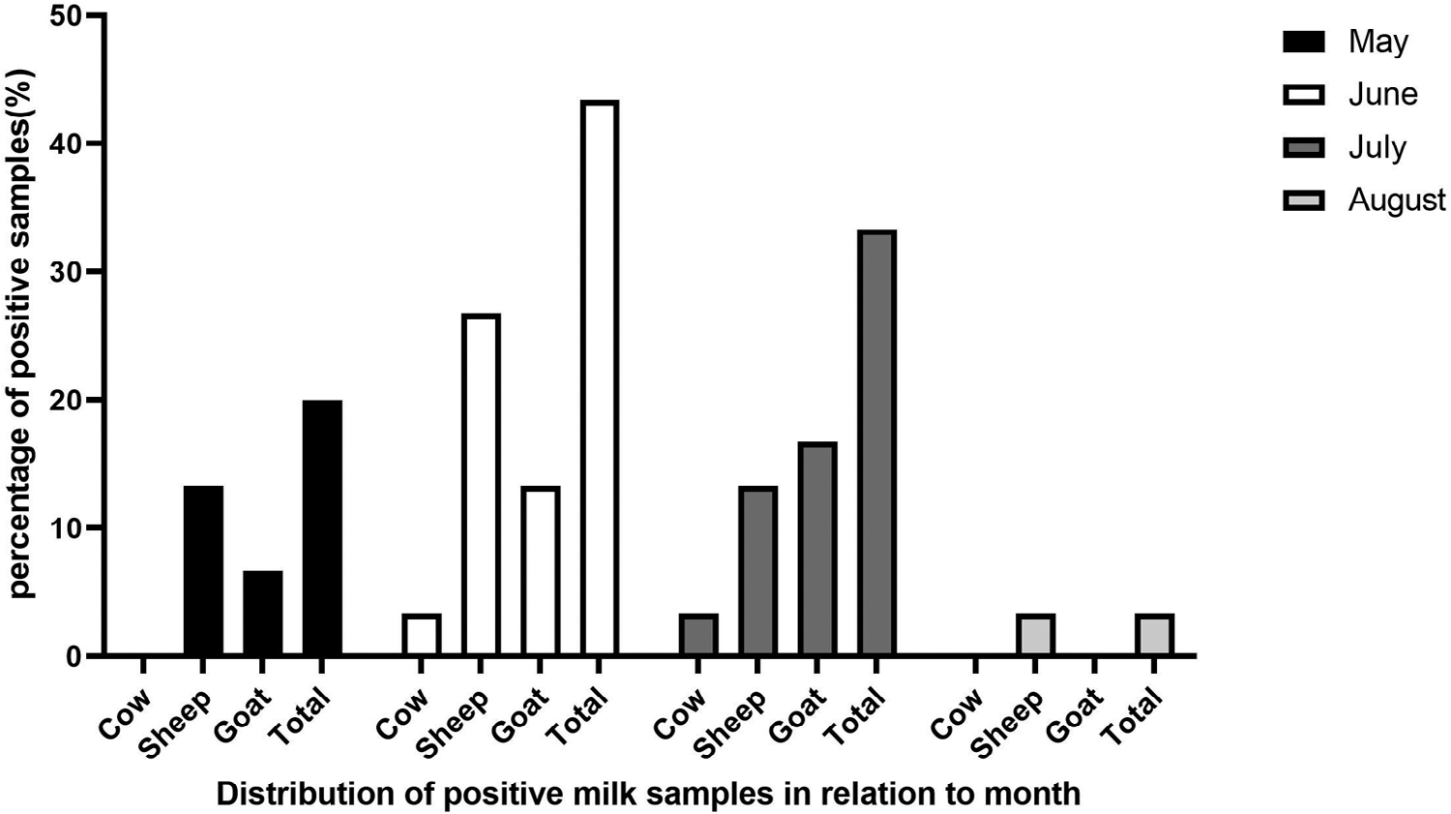
Distribution of positive milk samples in relation to month.

The examination of 180 unpasteurized milk samples from goats, cows and sheep collected from 7 farms (Table 3) by the nested RT‐PCR indicated the maximum positive rate of TBEV is in the milk of sheep (4.4%), which is similar to the prevalence of goat milk. On the other hand, the infection rate of TBEV in cow milk was (0%). All of the sequences revealed the strains belonged to the Sib subtype. An increase was observed in TBE infection at some of the sampling sites in June and July 2016 (Figure 2, Table 3). The viral loads of TBEV were determined using RT‐PCR in 16 positive samples. The median viral load in the positive samples was found to be 0.5 log 10 copies RNA/mL (0.4–0.6 log 10 copies RNA/mL). It was realized that the goat milk samples had higher viral loads than those of sheep and cows in the TBEV‐infected dairy animals. According to the ELISA experiment, the maximum infection rate was estimated in sheep milk (4.4%), then goat milk (2.2%) and cow milk (1.1%) (Table 4).

**TABLE 3:** Determination of the prevalence of tick‐borne encephalitis virus (TBEV) and antibodies in milk samples based on farm location and sampling day.

Animal species	Location	Sampling day	No. of samples	No. of positive samples by ELISA	No. of positive samples by RT‐PCR
Cow	ValaRud	05.17.2016	7	0	0
Sheep			7	2	2
Goat			6	1	1
Cow	Bulamaji	06.08.2016	9	0	0
Sheep			12	1	3
Goat			8	1	2
Cow	Nimavar	06.27.2016	6	1	0
Sheep			9	2	2
Goat			11	0	1
Cow	Ardin	07.09.2016	14	1	0
Sheep			7	3	1
Goat			7	0	2
Cow	Chiyar	07.29.2016	7	0	0
Sheep			8	0	0
Goat			10	1	2
Cow	Koushkan	08.08.2016	8	0	0
Sheep			6	1	0
Goat			9	0	0
Cow	Esfejin	08.27.2016	9	0	0
Sheep			11	0	0
Goat			9	0	0

Abbreviation: RT‐PCR, reverse transcription‐polymerase chain reaction.

**TABLE 4:** Prevalence of tick‐borne encephalitis virus (TBEV) in milk samples collected from different animal species, determined by reverse transcription‐polymerase chain reaction (RT‐PCR) and ELISA.

Method of detection	Animal species	Number of tested animals	Subtype	Total positive	(%)
ELISA	Cow	60	–	2	1.1
	Goat	60		4	2.2
	Sheep	60		8	4.4
	Total	180		14	7.7
RT‐PCR	Cow	60	Siberian	0	0
	Goat	60		8	4.4
	Sheep	60		8	4.4
	Total	180		16	8.8

## 4 DISCUSSION

This research marks the first documentation of TBEV transmission through unpasteurized milk in Iran. Our findings reveal that traditionally collected milk samples from pens tested positive for TBEV via both molecular and serological methods. Notably, all positive samples were identified as the Sib subtype, typically transmitted by *I. persulcatus* ticks. This aligns with reports from eastern Russia, where the Sib and FE subtypes are more common than the European subtype and may lead to more persistent or severe forms of the disease (Bonville & Domachowske, 2021; Ruzek et al., 2019; Volok et al., 2022).

The relevance of these findings is further underscored by the broader environmental changes impacting tick populations. Climate change has been known to affect the abundance of small mammals and the migration patterns of birds, which in turn influences the spread and activity of ticks, particularly in northern and humid areas (Waldenstrom et al., 2007; Wilhelmsson et al., 2020). *I. persulcatus* in northern Russia has a unimodal pattern of seasonal activity with a peak in May and June that declines during August (Bugmyrin & Bespyatova, 2023). During vegetation seasons, particularly at sufficient humidity and increased temperatures, these ticks are usually activated, with Zanjan province's related factors coming into play during the late spring to summer seasons (Solaimani et al., 2024). Investigations conducted by Borde et al. (2019), from 2001 to 2018, have proven that June and July had the highest frequency of TBEV infections. On the other hand, during cold seasons, the development and activity of these ticks are limited, as supported by a study conducted in Zanjan province on goats. The highest percentage of goat tick infestation was observed in June, whereas the lowest percentage was noted in February (Bahman Shabestari, 2010; Jafari et al., 2021). In alignment with their results, our research study also identified a very similar seasonal pattern.

In recent years, TBE infection rate has been rising as the virus has been spreading to new regions in various ways, such as through human travelling, birds’ migration, small mammals and climate change (Wondim et al., 2022). The spreading potential of infectious diseases through drinking unpasteurized milk in Iran was unknown until this study. The history of TBEV infection in domestic animals has not been studied comprehensively, even in endemic areas. Studies investigating domestic animals in response to suspected alimentary outbreaks have found that cows, sheep and goats show seropositivity. It is important to note that during the second phase of illness, the virus RNA result for milk samples is usually negative, whereas only antibodies can be detected in most cases (Pustijanac et al., 2023). This can be caused by the duration between the sampling of the suspected animals and the diagnosis of the human disease. Although it is generally expected that IgG antibodies to TBEV remain detectable for a longer period compared to the virus itself, our study presents a contrasting scenario. This can be attributed to two key factors: The rapid slaughter of animals, which likely restricted the full development of their antibody response, and the seasonal aspect of our sample collection, conducted during summer when TBEV is known to be more active (Blomqvist et al., 2021; Vilibic‐Cavlek et al., 2023). As a result, the virus is detectable primarily during the initial clinical phase, highlighting the importance of using both serological and molecular methods for accurate diagnosis and treatment (Balogh et al., 2010; Grešíková et al., 1975).

A few years after conducting our study, Salehi‐Vaziri et al. (2020) provided evidence of the circulation of TBEV in the rural population of northern Iran, which is humid with high temperatures during the hot seasons. An increasing number of human cases of TBE are emerging into new regions, not only in Iran but also throughout Europe and now in the Middle East (Panatto et al., 2022; Paquette et al., 2023; Salehi‐Vaziri et al., 2020). Wallenhammar et al. (2020) identified a new focus of TBEV in Örebro County, Sweden, through the monitoring of TBEV antibodies in milk. In Poland, a recent case of TBE linked to the consumption of consumption of unpasteurized goat's milk was documented, marking the fourth confirmed outbreak of TBEV infection in their country (Wójcik‐Fatla et al., 2023). In a study conducted by Ilic et al. (2020) in Croatia, TBEV testing using RT‐PCR on 12 goats from the implicated farm did not detect TBEV RNA in the milk. However, serological testing of goats and other farm animals revealed evidence of exposure to the virus, with six goats from the flock exhibiting TBEV‐neutralizing antibodies. Hay et al. (2016) found 9% positive for TBEV in milk in Kazakhstan.

Although many developed countries require pasteurized milk products, selling unpasteurized products is allowed in many regions of Iran (Haghi et al., 2015). Some people believe that raw milk has a better taste and has higher nutritional value than pasteurized milk. Many farming families may choose to consume raw milk for its natural state and lack of thermal processing (Offerdahl et al., 2016). Based on Salehi‐Vaziri et al. (2020) study on serological profiles in rural areas of Mazandaran province in Iran, the highest seropositivity was observed in occupations linked to farming and the animal industry, providing support for this assertion. More recently, raw milk vending machines in the dairy lands of Iran have been so available that many people are able to buy local unprocessed milk products. TBE transmitted by unpasteurized milk can be efficiently inhibited by vaccinating dairy animals or people (Henningsson et al., 2016); unfortunately, no vaccines are currently available for animals (Zimna et al., 2023), and the persistence of immunity against TBEV in animals has not been well understood yet (Salát et al., 2018). Hence, public health authorities should restrict farmers from selling milk without proper authorization and must educate the public about the dangers of consuming unpasteurized milk. They should highlight the benefits of boiling or pasteurizing milk before consumption or processing as a measure to reduce the risk of alimentary TBEV outbreaks.

## 5 CONCLUSION

This study found evidence of TBEV in Iran, particularly in the northern provinces. However, there were no reported cases of tick or dairy product contamination with TBEV during the study period, as diagnostic testing for TBEV is not routinely performed in Iran. The importance of this region and others in Iran warrants further investigation, especially given the preliminary positive serological findings in the northwestern part of Iran. This significance is underscored by the presence of TBE diseases in neighbouring countries indicating TBEV's geographical dispersion in specific regions can lead to the establishment of new foci. As TBEV spreads into new areas, further research is mandatory for detecting viral RNA in ticks and the human population. On the other hand, considering the sample collection occurred seven years ago, it is crucial to highlight that the dynamics of TBEV endemicity within the Zanjan province might have shifted since then. Several factors, including climate change, alterations in land use, variations in human activity and changes in the populations of ticks, can significantly affect the prevalence and geographic spread of the virus. Such changes could modify the risk landscape for TBEV transmission as identified in our study conducted in 2016. Consequently, although our research provides essential baseline data on the presence and distribution of TBEV for the first time in Iran during the period of study, it is imperative to conduct up‐to‐date research.

## AUTHOR CONTRIBUTIONS

*Conceptualisation; methodology; resources; software and data writing – original draft*: Angineh Parsadanians. *Data curation; funding acquisition; project administration; supervision and validation*: Hessam Mirshahabi. *Conceptualisation; methodology; supervision; validation and writing – review and editing*: Masoud Yavarmanesh.

## CONFLICT OF INTEREST STATEMENT

The authors declare that they have no known conflicts of interest or personal relationships that could have appeared to influence the work reported in this paper.

### PEER REVIEW

The peer review history for this article is available at https://www.webofscience.com/api/gateway/wos/peer-review/10.1002/vms3.1477.

## Data Availability

The data that support the findings of this study are available from the corresponding author upon reasonable request.
